# Supplementary material for: Solid-state laser refrigeration of a composite semiconductor Yb:YLiF4 optomechanical resonator
Source: Nat Commun. 2020 Jun 23;11:3235. doi: 10.1038/s41467-020-16472-6 (PMC7311430; doi:10.1038/s41467-020-16472-6)
Supplement: Supplementary file 1 — Supplementary Information [file 41467_2020_16472_MOESM1_ESM.pdf]

Supplementary information for "Solid-state laser refrigeration of a composite semiconductor Yb:YLiF$_4$ optomechanical resonator"

Anupum Pant[1], Xiaojing Xia[2], E. James Davis[3] & Peter J. Pauzauskie[1,4]

[1]*Materials Science and Engineering Department, University of Washington, Seattle, Washington 98195*

[2]*Department of Molecular Science & Engineering, University of Washington, Seattle, Washington 98195*

[3]*Chemical Engineering Department, University of Washington, Seattle, Washington 98195*

[4]*Physical & Computational Sciences Directorate, Pacific Northwest National Laboratory, Richland, Washington 99352*

Supplementary Note 1. Luminescence Thermometry

To use a direct measurement for obtaining the coldest temperature within the FCNR, differential luminescence thermometry (DLT) was used[1,2]. This approach involves fitting infrared emission of crystal field transitions of Yb(III) ions using a temperature-dependent Boltzmann distribution. Photoluminesnce (PL) spectra were collected and normalized with respect to the 960 nm peak ($E_6 \rightarrow E_1$), obtained from 10% Yb:YLF excited using irradiances between 0.039 and 0.965 MW cm^{-2}. The area under the dominant peaks representing the transitions $E_6 \rightarrow E_1$ (960 nm) and $E_5 \rightarrow E_3$ (993 nm) from Yb^{3+} ions in the YLF host have been labeled as I_1 and I_2, respectively. The temperature of the Yb:YLF was determined using a ratiometric analysis of the fluorescence spectra based on Boltzmann thermal population analysis of the truncated spectra by evaluating the ratio of areas under the curve I_1 and I_2 from the dominant peaks corresponding to the E_6 and E_5 transitions using the equation[1,2]:

$$\frac{I_1}{I_2} \propto \exp\left(\frac{-(E_6 - E_5)}{k_\mathrm{B}T}\right) \tag{1}$$

The natural log of the ratio (I_1/I_2) as a function of laser irradiance is calculated for spectra recorded at various laser irradiances. The decreasing value indicates cooling of the Yb:YLF crystal. The calibration was done by recording the ratio as a function of the cryostat temperature using a low laser irradiance of 0.039 MW cm^{-2}. A linear fit to the temperature data was used as a calibration to obtain temperature based on the value of the natural log of the ratio (I_1/I_2) measured at various irradiances.

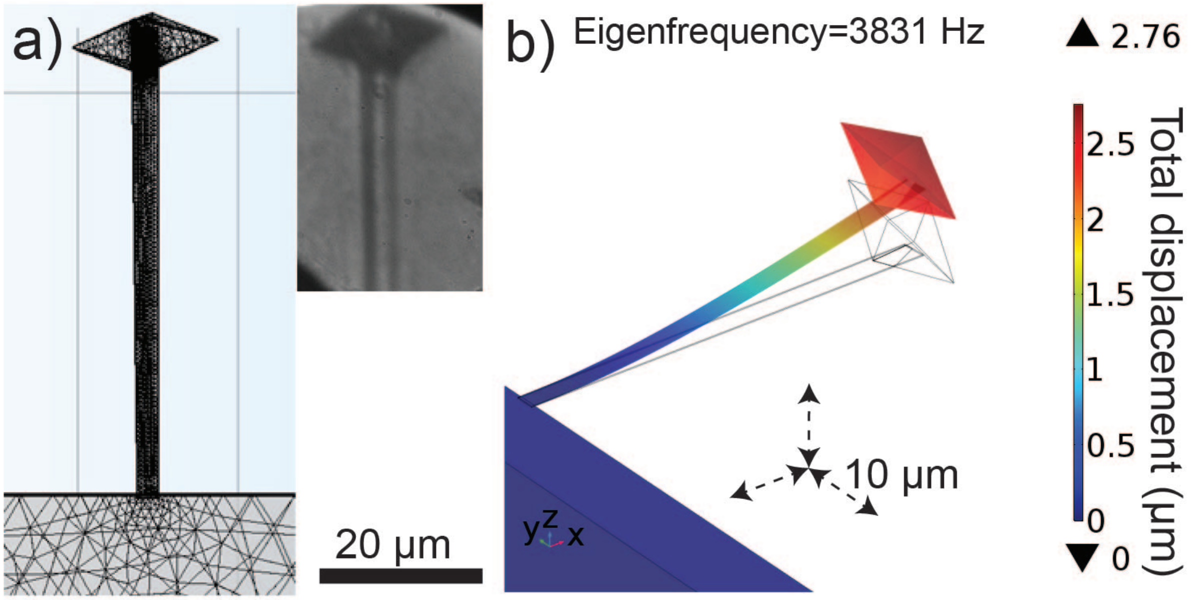

Supplementary Figure 1. Finite element model of the device. a) The finite element mesh model used for calculations is shown on the left and the optical image on the right shows the free end of 10% ytterbium doped lithium yttrium fluoride on a cadmium sulfide nanoribbon cantilever (FCNR). b) The results from the eigenfrequency modeling show an arbitrary displacement value at various coordinates across the FCNR for the first mode at 3831 Hz. A literature value for the Young's modulus (E) = 46.26 GPa[3], Poisson ratio (ν) = 0.3, and mass density ρ_{CdS} = 4826 Kg m^{-3} were used for cadmium sulfide. Mass density of Yb:YLF crystal was taken as ρ_{YLF} = 3890 Kg m^{-3}. The obtained eigenfrequency of the first mode agrees with the measured value of 3632.2 Hz.

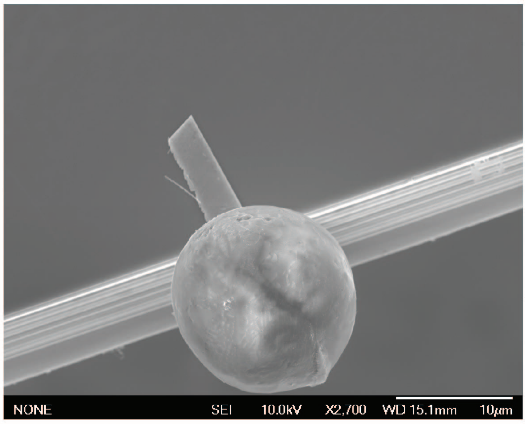

Supplementary Figure 2. Photothermal damage with 980 nm laser. SEM secondary electron image of a device destroyed due to overheating after 0.5 MW cm^{-2} of 980 nm irradiation. A CdS cantilever with a Yb:YLF crystal at the free-end was prepared and irradiated in vacuum with a 980 nm laser, using 0.5 MW cm^{-2} of irradiance. The Yb:YLF crystal instantaneously melted and morphed into a sphere. Consequently, the underlying cantilever melted and the particle receded away from the laser spot, towards the silicon substrate. The resulting device consisted of a melted sphere at the end of the remaining CdS cantilever base.

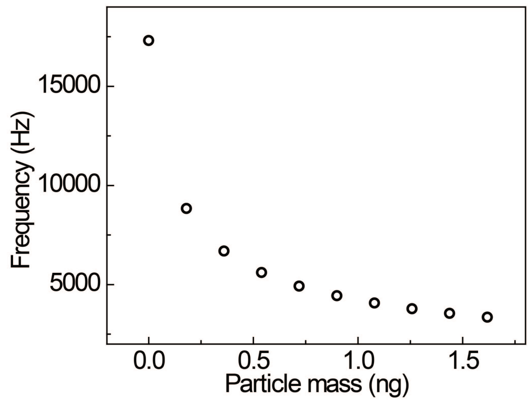

Supplementary Figure 3. Particle mass calculation. The eigenfrequency of the geometry in the finite element model was measured as the density of particle at the end of the cantilever was swept from 0 to 3890 Kg m^{-3} and the eigenfrequency with the mass of the particle is plotted. Finite element analysis shows that as the mass of the particle is increased from 0 to 1.36 ng at the free end, the eigenfrequency of the cantilever decreases from 17384 to 3632 Hz.

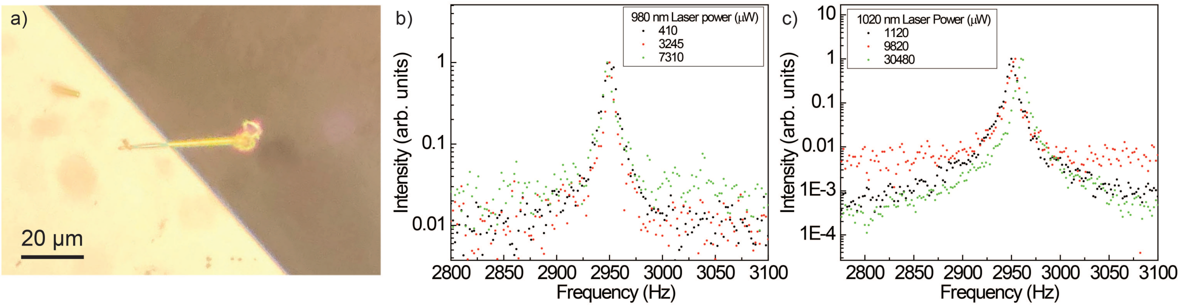

Supplementary Figure 4. Thermomechanical noise spectra with 980 and 1020 nm lasers. a) The optical image of the CdS device with a Yb:YLF crystal at the free end. b) Thermomechanical noise spectra near the fundamental mode eigenfrequency at different 980 nm laser powers c) and 1020 nm laser powers. The 980 nm irradiance was maintained below 0.2 MW cm^{-2} to prevent any damage to the device. The eigenfrequency of the device was obtained by measuring ten thermomechanical noise spectra at three different irradiances for each laser wavelength. The average peak position from a Lorentz fit to each of the 10 spectra was obtained for each laser power. The error bars denote one standard deviation.

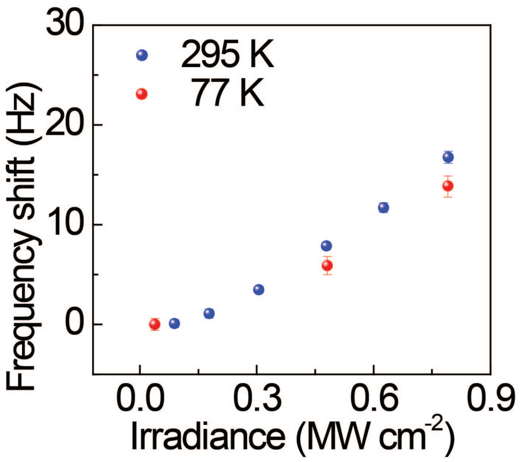

Supplementary Figure 5. Eigenfrequency measurements at 298 and 77 K. The shift in eigenfrequency of FCNR with increased irradiance measured at 298 and 77 K. The change in eigenfrequency at 77 K due to increasing irradiance is attributed to optical trapping forces, as the cooling of YLF is minimum due to the low efficiency of cooling resulting from the red shifting of mean florescence at low temperatures[4]. The difference of blueshift at room temperature, compared to that at 77 K (6 +/- 2.2 Hz at 40.1 mW) shown is therefore due to the laser refrigeration of the cantilever.

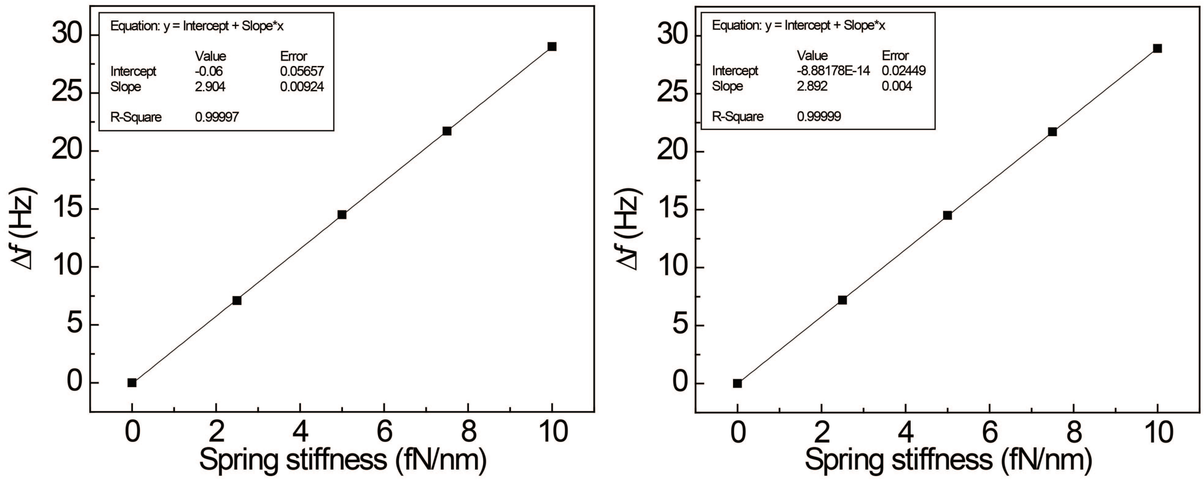

Supplementary Figure 6. Trapping stiffness simulations. The change in frequency of the cantilever modeled at a) room temperature and at b) 77 K to show the minimal difference even when both Youngs modulus and thermal expansion are accounted for. This shows the effect on the eigenfrequency due to the greater laser trap stiffness induced due to increasing irradiance is similar at 77 K and 298 K - 4.86 fN/nm is equivalent to 14.6 Hz of shift at both 77 K and 298 K. Thereby, allowing the deconvolution of laser cooling effect and laser trapping effect at room temperature by conducting the measurement of laser trap stiffness at low temperatures.

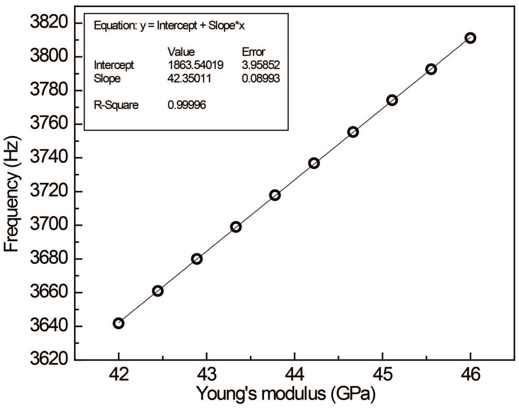

Supplementary Figure 7. Young's modulus dependent frequency simulation. The Young's modulus (E) of CdS was swept in the finite element model as described previously (Supplementary Figure 1), and the eigenfrequencies were calculated. To obtain the Young's modulus for CdS, a linear regression was fit to the parametric study and the Young's modulus required for the measured value of eigenfrequency, was derived. The linear fit to the simulation results gives a slope and intercept of 42.35 Hz GPa^{-1} and 1863.54 Hz, respectively.

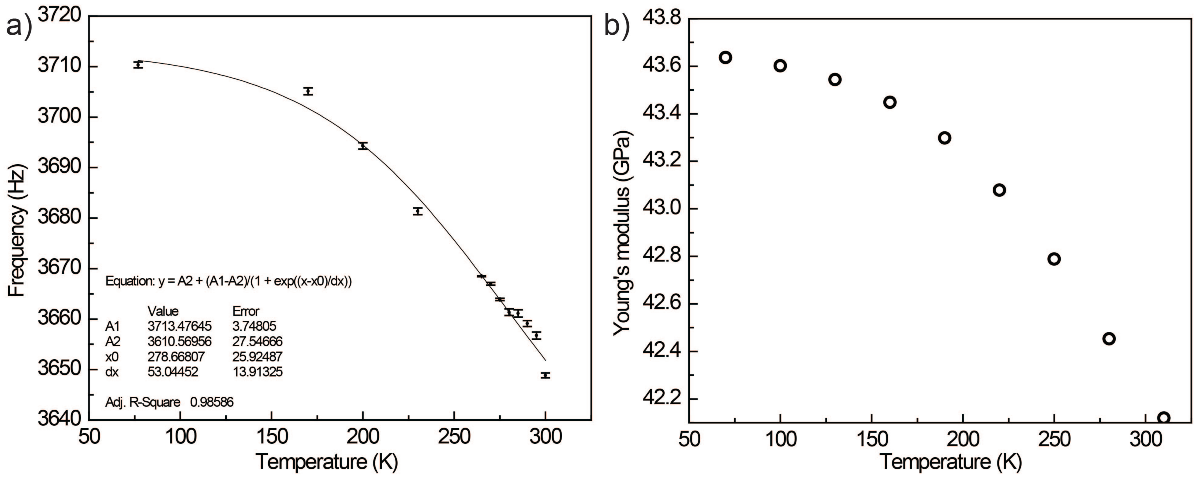

Supplementary Figure 8. Temperature dependent Young's modulus. a) Temperature calibration of the eigenfrequency of the FCNR done in the range of from 77 K to 300 K was fitted to the shown equation. The data points take into account the uncertainties in measurement by averaging the frequency value obtained from six thermomechanical noise spectra recorded at the given laser power. The error bars represent one standard deviation. b) The data from the eigenfrequency vs. temperature calibration and the eigenfrequency vs Young's modulus model obtained from finite element analysis was used to obtain a the change in Young's modulus with temperature. This is given as: E = 41.2529+((2.42991)/(1+exp((T-278.66807)/(53.04452)))) GPa

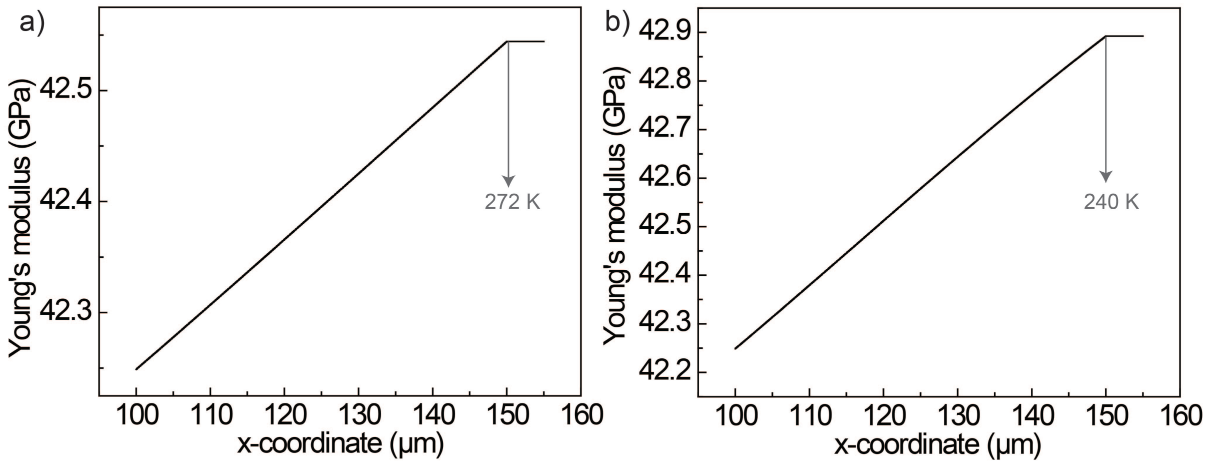

Supplementary Figure 9. Coordinate dependent Young's modulus. a) The gradient of temperature formed along the cantilever in steady state when a laser is focused near the tip is considered. Since the temperature calibration was done for an isothermal cantilever, the measured temperature change of -15.4 +/- 5.6 K would be an underestimate of the minimum temperature reached near the tip because, at high laser irradiances, a temperature gradient is established in the cantilever, where a spatially varying Young's modulus was considered to correlate the measured eigenfrequency values at isothermal conditions to the eigenfrequencies that exist in the context of a temperature gradient. It can be established that the minimum temperature near the Yb:YLF particle should be lower than the temperature measured using the isothermal calibration. To obtain the lowest temperature within the device using a combination of eigenfrequency measurement and finite element analysis, we model a spatially dependent Young's modulus. The Young's modulus distribution along the cantilever used to model the eigenfrequency has been shown when the tip is at a) 272 K and b) 240 K. The $\Delta f = f - f_0$ obtained is 3.8 and 8.1 Hz , respectively, where f_0 refers to the eigenfrequency obtained when the Young's modulus across the cantilever is 42.248 GPa (298 K).

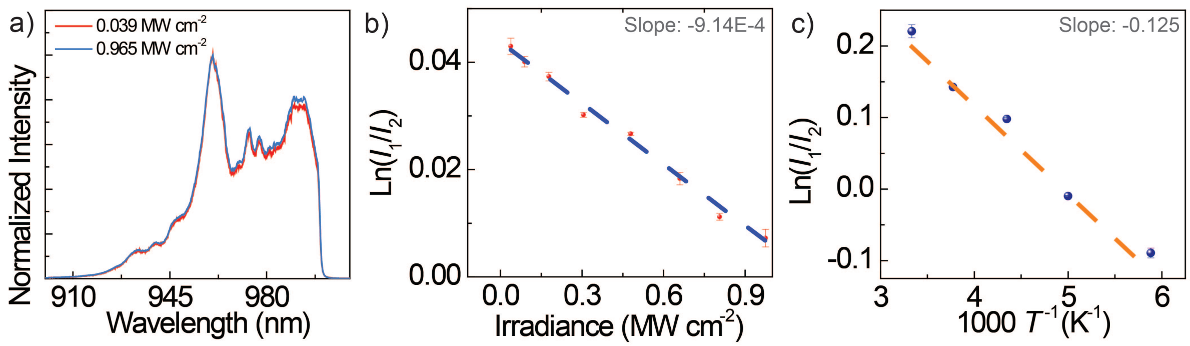

Supplementary Figure 10. Yb^{3+} Luminescence thermometry. a) Normalized up-conversion fluorescence spectrum using 0.039 (red) and 0.965 (blue) MW cm^{-2} irradiances of 1020 nm laser focused on the Yb:YLF crystal at the free end of the CdSNR cantilever. A 1000 nm short pass filter was used before the detector. The transitions E_6-E_1 and E_5-E_3, labeled as I_1 and I_2, respectively. b) The natural log of ratio of area under the curves in the region I_1 and I_2 shown in (a), as a function of the laser power c) and as a function of the cryostat temperature. Each data point was obtained by taking the mean of six values, each calculated from a distinct spectrum and the error bars represent one standard deviation.

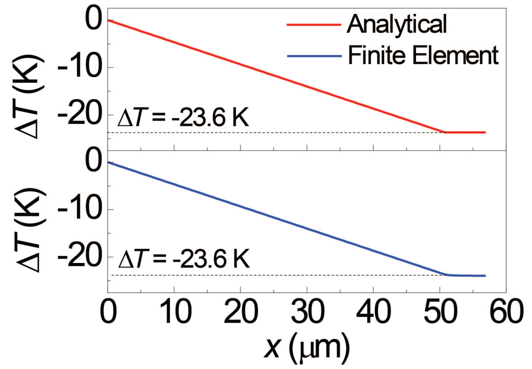

Supplementary Figure 11. Calculated temperature gradient. A comparison of temperature distribution obtained by an analytical model as described in the manuscript (red) with the temperature distribution obtained by finite element analysis. The 3D heat transfer model was solved numerically using finite element analysis software COMSOL. Excluding the heat source, the same geometry and parameters were used for analytical calculations. For finite element model, a constant heat source of 3.34x10^{-6} W over the volume of Yb:YLF was used. The temperature distribution was obtained and is shown in blue color. The temperature gradient obtained from both the models overlap.

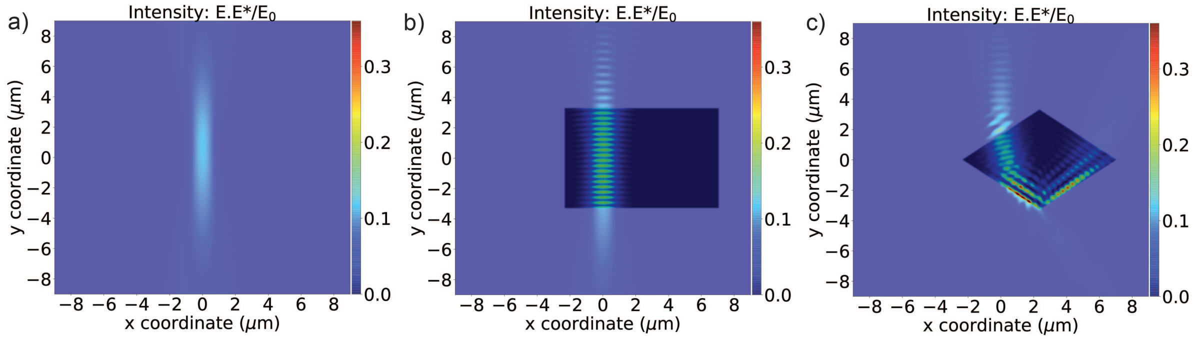

Supplementary Figure 12. Finite difference time domain simulations. The MIT Electromagnetic Equation Propagation (MEEP) software package implemented in PYTHON was used to perform a 2D finite-difference time-domain (FDTD) simulation of a focused 1020 nm Gaussian laser beam incident on various geometries. The total intensity normalized to the incident electric field ($\mathbf{E} \cdot \mathbf{E}^{*}/\mathbf{E_0}$) are plotted over. a) Free-space b) Rectangle and c) Bipyramid cross section. A clear enhancement and multiple internal reflections of the pump beam can be seen within the bipyramid cross section. Quantitatively, the maximum optical intensity within the bipyramid (0.36) cross section is two times of that within the rectangular shape (0.18).

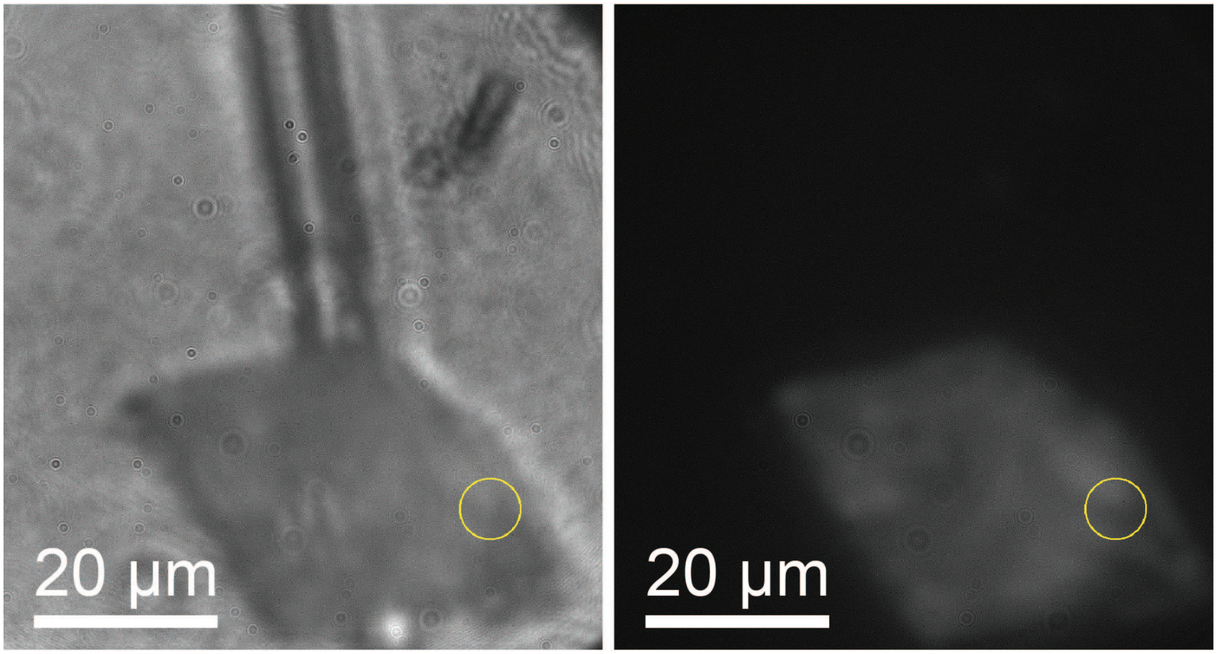

Supplementary Figure 13. Fluorescence image of Yb:YLF. a) A bright-field optical image of the free-end of a CdS cantilever with a large Yb:YLF crystal recorded with the lamp light switched on. A 1020 nm laser was focused to a spot size shown using a yellow outline and the fluorescence was imaged using a 1000 nm short-pass filter in front of the CCD camera to remove any scattered 1020 nm laser light. b) Optical image of the crystal without the lamp light, as one end of the crystal is irradiated with the laser at the spot indicated using the yellow outline. Fluorescence was imaged using a 1000 nm SP filter to remove the scattered 1020 nm laser light. It is evident from the fluorescence image that due to the multiple internal reflections of the pump laser, the fluorescence is excited nearly evenly across the crystal even when only a small part of the crystal is irradiated.

Supplementary References

1. Melgaard, S. D., Albrecht, A. R., Hehlen, M. P. & Sheik-Bahae, M. Solid-state optical refrigeration to sub-100 kelvin regime. *Sci. Rep.* **6**, 20380 (2016).

2. Rahman, A. A. & Barker, P. Laser refrigeration, alignment and rotation of levitated Yb^{3+}: YLF nanocrystals. *Nat. Photonics* **11**, 634–638 (2017).

3. Gerlich, D. The elastic constants of cadmium sulfide between 4.2–300° K. *J. Phys. Chem. Solids* **28**, 2575–2579 (1967).

4. Seletskiy, D. V. *et al.* Precise determination of minimum achievable temperature for solid-state optical refrigeration. *J. Lumin.* **133**, 5–9 (2013).

Acknowledgements A.P., X.X., and P.J.P. gratefully acknowledge financial support from the MURI:MARBLe project under the auspices of the Air Force Office of Scientific Research (Award No. FA9550-16-1-0362). Sample characterization was conducted at the University of Washington Molecular Analysis Facility, which is supported in part by the National Science Foundation (Grant No. ECC-1542101), the University of Washington, the Molecular Engineering & Sciences Institute, the Clean Energy Institute, and the National Institutes of Health.

Competing Interests The authors declare that they have no competing interests.

Correspondence Correspondence and requests for materials should be addressed to Peter J. Pauzauskie. (email: peterpz@uw.edu).
